# Rare Codons Cluster

**DOI:** 10.1371/journal.pone.0003412

**Published:** 2008-10-15

**Authors:** Thomas F. Clarke, Patricia L. Clark

**Affiliations:** Department of Chemistry & Biochemistry, University of Notre Dame, Notre Dame, Indiana, United States of America; University of Hong Kong, China

## Abstract

Most amino acids are encoded by more than one codon. These synonymous codons are not used with equal frequency: in every organism, some codons are used more commonly, while others are more rare. Though the encoded protein sequence is identical, selective pressures favor more common codons for enhanced translation speed and fidelity. However, rare codons persist, presumably due to neutral drift. Here, we determine whether other, unknown factors, beyond neutral drift, affect the selection and/or distribution of rare codons. We have developed a novel algorithm that evaluates the relative rareness of a nucleotide sequence used to produce a given protein sequence. We show that rare codons, rather than being randomly scattered across genes, often occur in large clusters. These clusters occur in numerous eukaryotic and prokaryotic genomes, and are not confined to unusual or rarely expressed genes: many highly expressed genes, including genes for ribosomal proteins, contain rare codon clusters. A rare codon cluster can impede ribosome translation of the rare codon sequence. These results indicate additional selective pressures govern the use of synonymous codons, and specifically that local pauses in translation can be beneficial for protein biogenesis.

## Introduction

A synonymous DNA mutation will alter the nucleotide sequence but, due to the degeneracy of the genetic code, does not alter the encoded amino acid sequence. Hence, a synonymous mutation is less likely to affect protein function than a non-synonymous mutation. Yet even synonymous mutations are not entirely neutral: there is a weak selection for synonymous codons that are more common [1]. Which codons are more common varies by organism [2], and is determined by a wide variety of factors, including GC bias. The weak selection for common codons is thought to occur primarily because common codons are translated more quickly (providing more regulatory control) and with higher fidelity (producing more accurate protein sequences) than rare codons [3]. Highly expressed genes are therefore enriched with common codons [4]. The persistence of rare codons is attributed to neutral drift [5].

Previous studies of codon usage used algorithms designed to highlight common codons, not rare codons [6], [7]; this reflects the general interest in increasing translation rate to improve protein expression levels, regardless of the effect on folding yield. The mathematics underlying these algorithms is therefore not designed to highlight the frequency and distribution of rare codons. Many previous studies of the distribution of rare codons [8], [9] examined only the absolute usage frequency of any one codon versus all 63 other codons, and detected no strong evolutionary pressure on synonymous codon selection. But an absolute comparison of codon usage frequency can not take into account the evolutionary pressure to maintain a given amino acid residue at a particular position, for example for protein folding, stability, and/or function. Furthermore, studies that rely on cellular tRNA concentration alone as an indicator of translation speed [10] are subject to the caveat that the speed of translation can vary for different codons that use the same tRNA [11]. Since the major influence of codon usage is on local translation rate, a more complete understanding of the impact of codon usage on translation rate could assist in optimizing protein expression to maximize protein yield *in vivo*, interpreting *in vitro* folding pathways, and predicting protein domains *in silico*. Here, we use a novel approach to investigate whether additional selective pressures play a role in synonymous codon usage.

## Results and Discussion

**Figure 1:**
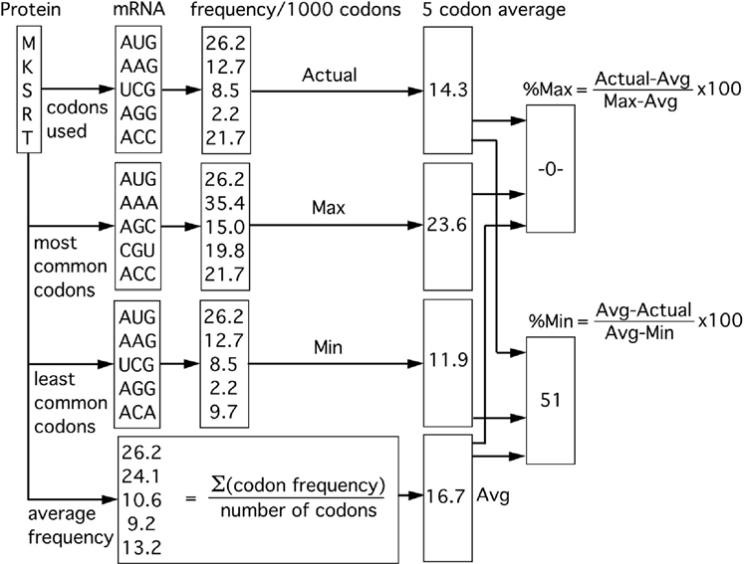
%MinMax analysis for the pentapeptide MKSRT, encoded by AUGAAGUCGAGGACC (total number of codons per amino acid: M, 1; K, 2; S, 6; R, 6; T, 4). For each codon, three *E. coli* absolute codon frequencies are tabulated using codon usage data from KazUSA [26]: (*i*) the frequency with which this codon is used in the entire *E. coli* genome (Actual), (*ii*) the usage frequency for the most common codon encoding this amino acid (Max), and (*iii*) the usage frequency for the least common codon encoding this amino acid (Min). An average usage frequency (Avg) is also calculated for each residue by summing the individual codon frequencies and dividing by the number of codons (for each residue). The resulting values are typically averaged over an 18-codon window (a window of 5 is used here); window sizes from 5 to 30 codons produced similar distributions of rare codon clusters, though the noise was increased with smaller window sizes. These four codon usage frequencies are used to calculate %Max and %Min using the equations shown; note that only positive values are reported (i.e., each window may yield a value for either %Min or %Max, not both). A %Min value of 51 means that this sequence is approximately halfway between the maximum rare sequence and the average sequence, and is plotted as −51.

**Figure 2:**
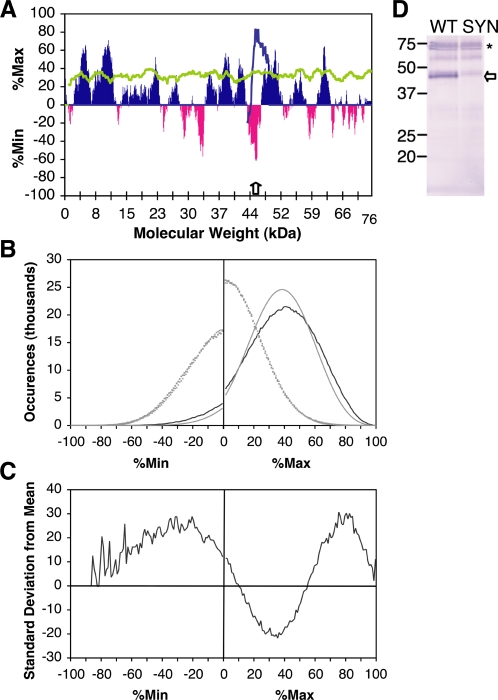
Codon clustering in bacterially expressed genes. (A) %MinMax was applied to the P22 tailspike gene, using a sliding window size of 18 codons and the *E. coli* codon bias (essentially identical to the codon bias of *S. enterica* serovar *Typhimurium*, the endogenous host of P22). Dark %Max bars correspond to clusters of common codons; lighter %Min (negative) bars correspond to clusters of rare codons. In contrast, the average of 200 random reverse translations of tailspike, biased to *E. coli* codon usage frequencies, yields a %MinMax profile that is entirely %Max (grey line). The white arrow marks the location of the deepest %Min peak, at codon 406. Silent mutagenesis of P22 tailspike to replace this rare codon cluster with synonymous common codons alters the %MinMax plot (black line); these mutations only affect the indicated %Min peak. (B) The %MinMax value for every window of the entire *E. coli* ORFeome was calculated using a sliding window of 18 codons and used to construct a histogram of %MinMax values at intervals of 1%MinMax. Negative bin numbers represent %Min values. The effects of codon clustering are seen when the *E. coli* ORFeome (black line) is compared to the +1 and −1 out-of-frame sequences of the *E. coli* genome (dotted lines) or the average of 200 codon-biased random reverse translations analyzed using the same statistical conditions as the entire ORFeome (grey line). (C) The deviation of the distribution of %MinMax bins throughout the *E. coli* ORFeome from the average of 200 codon-biased random reverse translations of the entire ORFeome is greatest in high %Max regions (30 standard deviations from mean), and at −31%Min (28 standard deviations from mean). (D) Tailspike was expressed *in vivo* on *E. coli* ribosomes. After lysis, the N-terminal His-tag of tailspike was detected using an anti-His tag antibody, revealing two major bands: full length tailspike (asterisk), which dwells on the ribosome post-translationally [27], and a 49 kDa band corresponding to the size of a nascent chain produced during pausing at approximately codon 406, the location of the deepest %Min peak (white arrow). Silent mutagenesis to eliminate the large rare codon cluster centered at codon 406 (SYN) eliminates the 49 kDa band.

In order to determine the relative rareness of the codons used to encode a particular amino acid sequence, we developed the %MinMax algorithm. %MinMax defines the relationship between a given mRNA sequence and hypothetical sequences encoding the same protein using the most rare (minimum) or most common (maximum) codons, as a function of the arithmetic mean of all possible codon usage frequencies. The complete %MinMax algorithm is shown in Methods; Figure 1 illustrates %MinMax calculations for a pentapeptide encoded with *E. coli* codon usage frequencies. A sliding window of %MinMax output along an mRNA sequence produces a plot in which clusters of predominantly common codons appear as positive (%Max) peaks, and clusters of predominantly rare codons appear as negative (%Min) peaks (Fig. 2A). A value of −100% represents a sequence window encoded using only the most rare codons, while a value of 100% represents a sequence encoded using only the most common codons. A value of 0% represents codon usage equal to the mean of all possible codon choices for a given amino acid sequence. For example, a window of 18 codons containing 9 of each of the two histidine codons would result in a 0% value.

We applied the %MinMax algorithm to all *E. coli* open reading frames (ORFs) [12] in order to determine the frequency and distribution of rare codons. As expected, based on the genome-wide selection for common codons [1], the ORFeome is highly populated with strongly %Max windows (Fig. 2B). Yet surprisingly, the number of strongly %Max windows in the ORFeome is significantly (30 standard deviations) larger than the number expected by random selection of codons within the constraints of the *E. coli* codon bias (Fig. 2C). In other words, there are many more windows than expected where rare codons are avoided, even when codon bias is included. Clusters of rare codons (%Min windows) are also over-represented, up to 28 standard deviations from the mean at the point of greatest deviation from the average for random codon selection (−31%Min) (Fig. 2C); i.e., windows containing rare codons are significantly enriched in the *E. coli* ORFeome. These windows are retained regardless of the window size selected (from 10 to 30 codons), although for larger window sizes, the point of highest deviation from the mean shifted to lower %Min values (−27%Min for a window size of 30 codons). On average, larger window sizes are more likely to introduce additional common codons, lessening the severity of a rare codon cluster.

**Figure 3 pone-0003412-g003:**
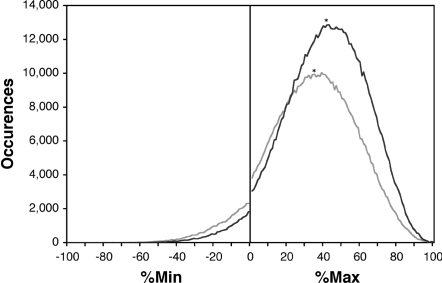
Codon clustering within subsets of the *E. coli* ORFeome, separated by gene classification. 2166 characterized genes from the *E. coli* ORFeome (dark line) are enriched in common codons as compared to 2325 genes annotated as unclassified, hypothetical, or unknown function (grey line). The median of each curve is denoted with an asterisk. %MinMax values were calculated using the codon usage frequencies from the entire ORFeome, with a sliding window of 18 codons.

Separating the ORFeome into assigned genes versus uncharacterized and hypothetical genes showed that rare codon clusters are not exclusive to uncharacterized and hypothetical genes, although there is a slight enrichment of rare codons in these genes (Fig. 3). Moreover, when the %MinMax algorithm was performed on +1 or −1 out-of-frame codons, the codon usage distribution was centered near average (%Min = %Max = 0) (Fig. 2B, dotted lines), indicating that the distribution reported for the in-frame ORFs is not simply a product of the algorithm itself, or a genomic artifact.

Ranking *E. coli* genes according to both the overall frequency of %Min windows and the magnitude of the largest %Min window identified 1024 genes (of 4288 total) with at least one %Min window deeper than −30%Min, the point of maximum standard deviation from the mean for *E. coli*. Moreover, there are 80 *E. coli* genes with at least one %Min window deeper than −60%Min. Furthermore, most of these 80 genes have few overall %Min windows, indicating significant rare codon clusters appear in many genes that primarily use common codons (ribosomal protein L5 is one example). This is surprising, given that common codons are an indicator of high expression [6], which should be negatively affected by a rare codon cluster [3].

**Figure 4:**
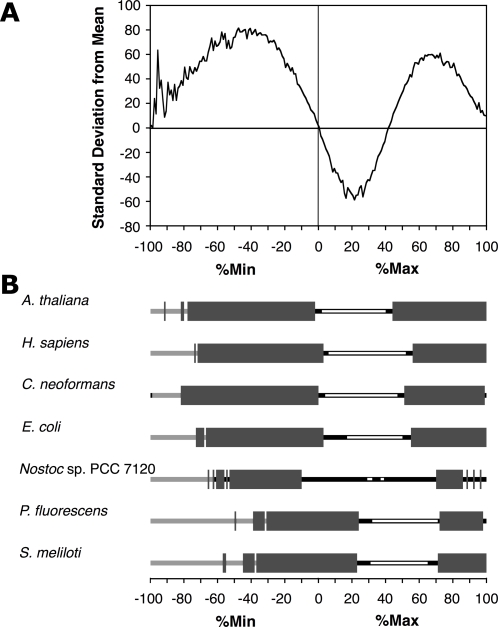
Codons cluster in a wide variety of organisms. (A) The %MinMax distribution for every gene of the *Arabidopsis thaliana* genome annotation database was calculated using a window size of 18 codons and compared to 200 random reverse translations as described for Figure 2B. *A. thaliana* shares a similar enrichment of rare codon clusters and very common codon clusters as seen for the *E. coli* ORFeome (Figure 2B). (B) A wide variety of organisms are enriched for rare and very common codon clusters. Regions of enrichment (≥8σ from the mean, thick grey bars) were observed for the ORFeomes of eukaryotes *A. thaliana*, *H. sapiens*, and *C. neoformans*, as well as prokaryotes *E. coli*, *Nostoc*, *P. fluorescens* and *S. meliloti*. The low %Max regions, which represent a more random distribution of rare and common codons (less clustering), were typically either significantly under-represented (open bars) or not significantly different from the random reverse translations (black bars). In some extreme regions, the random reverse translations were unable to provide sufficient coverage to ensure a normal distribution of the data (light grey bars); see Methods for more details.

Analyses of ORFeomes from 24 other prokaryotic organisms revealed similar distributions of codon clustering, regardless of GC content. The ORFeomes of *Nostoc* sp PCC 7120 (42%GC), *Pseudomonas fluorescens* (63%GC) and *Sinorhizobium meliloti* (63%GC) all have similar enrichment of both rare and extremely common codon clusters to the results for *E. coli* (52%GC) (Fig. 4), demonstrating that codon clustering is not limited to a particular genotype profile. Of the 25 prokaryotic ORFeomes examined (see list in Methods), 22 returned statistically significant (≥8σ) enrichment of rare codon clusters. Furthermore, analyses of ORFeomes from nine diverse eukaryotic organisms also revealed a similar distribution of codon clustering. Indeed, all eukaryotic ORFeomes examined, including *Homo sapiens*, *Arabidopsis thaliana*, and the fungus *Cryptococcus neoformans*, show enrichment in %Min windows, as well as %Max windows higher than 70%Max (Fig. 4). Enrichment of rare codon clusters in such a broad range of organisms suggests a general evolutionary selection pressure for clustering, despite the established negative effects on local translation rate.

As mentioned above, our analysis revealed distinct, deep %Min peaks, corresponding to clusters of rare codons, in many highly expressed genes, including the *Salmonella* phage P22 tailspike protein (Fig. 2A). To determine the effects, if any, of these rare codon clusters on tailspike translation, *E. coli* cells over-expressing N-terminally His-tagged tailspike were analyzed for translational pauses. During over-expression, the distribution of tailspike chain lengths was assayed by western blotting (Fig. 2D). In addition to full-length tailspike, a shorter fragment, corresponding to the size of a nascent tailspike chain attached to a ribosome paused at the deepest tailspike %Min window (Fig. 2A, arrow), was also detected. Silent mutagenesis to eliminate this deepest tailspike rare codon cluster also eliminated the corresponding tailspike band (Fig. 2D, arrow).

While there is a substantial body of literature on the negative effects of rare codons on protein production [3], there have also been reports of potential positive effects of rare codons on protein biogenesis [7], [13]–[16], including conserved rare codons [17]. Intriguingly, two recent studies have highlighted isolated rare codons that increase protein activity, either via higher expression levels (derived from increased mRNA stability) [18], or an altered native conformation (perhaps derived from modified co-translational folding) [19].

The significant clustering of rare codons reported here suggests there is strong selective pressure to maintain rare codon clusters in a wide variety of genes, across a broad range of organisms, and runs counter to the assumption that synonymous codon substitutions are essentially genomic background noise [20]. The major influence of codon usage is on local translation rate, and large clusters will have a greater effect on protein production than an equivalent number of randomly scattered rare codons [21], [22]. Reports of improved folding yield or protein activity due to translational pausing (reviewed in [23], [24]) highlight potential factors that might lead to the enrichment of rare codon clusters. These results have implications for the role of rare codon clusters in all aspects of protein expression: mRNA stability, folding, secretion, and interactions with partner proteins.

## Materials and Methods

### %MinMax algorithm

For the *j*th codon of the *i*th amino acid with *n* synonymous codons, the %MinMax algorithm (described schematically in Fig. 1) calculates the difference between the actual codon usage frequency (X*_ij_*) and the average codon usage frequency (X*_avg,i_*), divided by the difference between the maximum (X*_max,i_*) or minimum (X*_min,i_*) codon usage frequency and the average codon usage value:
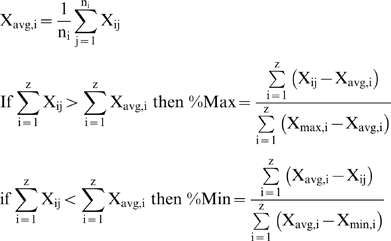
The codon frequency X is determined over a sliding window of *z* codons; all results shown in Figures 2–4 used a window size of 18. The output of each %MinMax equation is, by definition, always positive. If the codon usage frequencies for a given window are greater than the average, a value will be returned for %Max; if it is less than the average, a value will be returned for %Min. For clarity, %Min values are plotted and reported as negative numbers. For any sequence, the %MinMax output is presented as a series of sliding windows (1 to z, 2 to z+1, etc.). Specific codon positions reported in the text represent the midpoint of the window (for example, “406” represents the window encompassing codons 397–414).

### Computational methods

The *E.coli* K12-MG1655 ORFeome, containing 4288 ORFs, was obtained from the TIGR CMR database [12]. The ORFeomes of *Cryptococcus neoformans*, *Nostoc* sp PCC 7120, *Pseudomonas fluorescens* and *Sinorhizobium meliloti* were also obtained from TIGR; the ORFeome of *H. sapiens* was obtained from the DFCI-CCSB at Harvard [25]. These ORFeomes were chosen as a representative set, comprising a wide-range of GC bias as well as four separate taxonomic kingdoms. The remaining prokaryotic ORFeomes (*Agrobacterium tumefaciens*, *Bacillus anthracis*, *Bacillus cereus*, Bacillus subtilis*, *Bacteriodes fragilis*, *Bordetella pertussis*, *Brucella melitensis 16M*, *Burkholderia sp. 383*, *Coxiella burnetii*, *Deinococcus radiodurans*, *Erwinia carotovora*, Heliobacter pylori*, *Neisseria meningitidis*, *Ralstonia metallidurans CH34*, *Salmonella entericia*, *Salmonella typhimurium*, *Shigella flexneri*, Staphylococcus aureus*, *Thermus thermophilus*, *Xylella fastidiosa* and *Yersinia pestis*) were obtained from the TIGR CMR. The three ORFeomes that did not show statistically significant clustering of rare codons are marked with an asterisk (*). The remaining eukaryotic datasets (*Aspergillus fumigatus*, *Brugia malayi*, *Entamoeba histolytica*, *Neosartorya fischeri*, *Plasmodium yoelli*, *Theileria parva*, *Trypanosoma brucei*) were obtained from their respective genome projects at TIGR.

Codon usage frequencies were calculated directly from the ORFeome of each organism. All windows that contained a non-ATGC base were eliminated. %MinMax analyses of individual genes and codon-biased random reverse translations, including statistical analyses, were performed using Perl. GC content was calculated from the observed codon populations within the respective database. For calculations involving *E. coli* hypothetical genes, ORFs annotated in the TIGR CMR database as hypothetical, conserved hypothetical, or unclassified were pooled and compared to characterized ORFs.

Codon-biased random reverse translations were performed by substituting the wild type codon with a codon encoding the same amino acid randomly selected from a look-up table weighted for codon usage frequency. The mean and standard deviation of the %MinMax output for the random reverse translations was compared to the actual number of observances. To ensure the data was normally distributed, the percentage of data points localized within 1 through 4 standard deviations was determined and compared to the ideal 68.2, 95.5, 99.7, and 99.99% distributions. %MinMax values for which the sum of the difference from the ideal distribution exceeded ten percent were determined to be not normally distributed; this assignment occurred primarily in the extreme %Min region, where populations of random reverse translations would be required to contain almost exclusively the rarest codon for each amino acid, a statistically unlikely event.

### Experimental methods

*E. coli* BL21(DE3)pLysS transformed with a plasmid expressing either wild-type or pause-deleted His-tailspike were grown to an OD_600_ of 0.4, and protein expression was induced with 1 mM IPTG for 2 h. Chloramphenicol (250 µg/mL) was added to arrest translation. Cells were harvested and boiled in SDS gel-loading buffer, separated by SDS-PAGE, and the N-terminal His-tag was detected by Western blot using an anti-His antibody (Invitrogen).
